# Situational analysis and reflections on the introduction of advanced practice nurses in Brazilian primary healthcare

**DOI:** 10.1186/s12960-021-00632-w

**Published:** 2021-07-22

**Authors:** Carinne Magnago, Celia Regina Pierantoni

**Affiliations:** 1 Department of Policy, Management and Health, School of Public Health, University of São Paulo (FSP/USP), Av. Dr. Arnaldo, 715, São Paulo, SP 01246-904 Brazil; 2 Institute of Social Medicine, Rio de Janeiro State University (IMS/Uerj), Rio de Janeiro, Brazil

**Keywords:** Advanced practice nursing, Primary health care, Scope of practice, Nurse's role, Baccalaureate nursing education, Nursing legislation, Brazil

## Abstract

**Background:**

The shortage of doctors and their unequal distribution serve as challenges to advancing primary healthcare (PHC) and achieving effective universal healthcare coverage in Brazil. In an effort to use nurses’ potential more efficiently, the country is investigating the introduction of the advanced practice nurse (APN) into PHC. This paper presents a situational analysis of the practices of Brazilian nurses based on the following components: regulation, practice, and education.

**Methods:**

This is a national multi-method study with triangulated data from a documentary study, a scoping review, and an exploratory study. The regulation component involved the analysis of official normative documents on the regulation of nursing education and nurses’ scope of practice. The practice component aimed to identify the practices performed by nurses in Brazilian PHC based on primary studies. The education component intended to identify the practices taught in nursing training based on a survey and interviews with directors of undergraduate nursing programs.

**Results:**

Federal legislation in Brazil authorizes nursing graduates to perform a set of advanced practices as part of the PHC nurse's daily routine. They can request and interpret complementary tests and prescribe medication. However, in the local context, municipalities define the scope of this assistance based on technical norms or nursing protocols. Furthermore, this study indicates that undergraduate nursing programs do not fully prepare students to adequately execute these tasks.

**Conclusions:**

In the context of Brazilian PHC, advanced practices have already been implemented and respond to main healthcare demands. Therefore, it is unnecessary to introduce the APN as a new professional category. Upon detecting deficiencies in the training process, the current education model should undergo reforms that seek to incorporate the skills compatible with the regulated advanced practices and in-service training for practicing nurses. Regarding the introduction of APN along international lines, this article presents recommendations that may support the operationalization of a Brazilian APN model.

**Supplementary Information:**

The online version contains supplementary material available at 10.1186/s12960-021-00632-w.

## Background

Brazil is a country with continental dimensions that is subdivided into five geographic macroregions (North, Northeast, Midwest, Southeast, and South) with different demographic and socioeconomic conditions and wide internal inequalities [1]. The Brazilian Unified Health System (SUS), that is oriented toward primary healthcare (PHC), suffers from a shortage, high turnover, and unequal distribution of physicians [2, 3], especially in the rural and remote areas of the Northern and Northeastern macroregions. This threatens the population's access to high-quality, definitive care [4, 5]. Approximately 20,000 doctors graduate annually in Brazil, and they are mostly incorporated into private healthcare services in large urban centers [5–7]. Meanwhile, there is a growing number of nurses in the labor market (40,000/year), with greater availability to work in the public healthcare sector, whose services could be more efficiently utilized [6, 7]. New profiles such as the advanced practice nurse (APN) may be instrumental in advancing Brazilian PHC and achieving universal healthcare coverage [8].

The APN has specialized knowledge, complex decision-making skills, and clinical skills for a broader scope of practice, and their characteristics are shaped by the context in which they are certified to practice [9–12]. In PHC, the APN operates with an expanded scope of practice that incorporates the physician’s tasks and involves either substituting or complementing the physician’s work [9]. In the United States and Canada, advanced practices include diagnosing and treating acute and chronic diseases, ordering and interpreting diagnostic tests, prescribing drugs without a physician’s supervision, and referring patients to specialists [9, 13, 14]. Mirroring the experiences of these countries, Brazil is investigating the possibility of introducing the APN into primary care [15].

Given the incipience of national studies that are oriented toward the implementation of the APN in the SUS, this study analyzes the practices of Brazilian nurses based on three components—regulation, practice, and education—to identify and compare the nature of authorized practices, practices developed in PHC, and practices taught in undergraduate nursing programs.

## Methods

We conducted a national multi-method study that triangulated primary and secondary data, in order to analyze the problems, conditions, and opportunities involved in introducing APN in Brazil. For the regulation component, we analyzed a set of official normative documents that detail the regulation of nursing education and the nurse’s scope of practice in the context of PHC. We retrieved these documents in March 2021 from the websites of the Ministries of Health and Education and the Federal Nursing Council (COFEN)^1^ and conducted a qualitative analysis of their content.

For the practice component, we performed a scoping review [16] of primary studies conducted on nurses practicing in PHC in Brazil to identify the practices effectively performed by these professionals. In March 2021, we used a standard search strategy to consult different scientific databases, and 22 of the 780 studies found were selected for analysis. This set of studies included 3618 PHC nurses and described more than 90 activities performed in daily professional practice (More information can be found in Additional File 1). We presented the results of the review in the form of a narrative synthesis.

We examined the education component based on a national exploratory study that conducted telephone surveys and face-to-face interviews with directors of undergraduate nursing programs; some of these data have been previously published [17–19]. In this study, we assessed the data that revealed the specific practices that nursing programs had taught their students. The telephone surveys were conducted with 94 directors in the first semester of 2016, after a sample calculation based on the total number of programs in 2013 [17]. We used descriptive statistics to analyze the data. Between November 2015 and March 2017, face-to-face interviews were conducted with 16 directors who did not participate in the telephone survey [18]. The interviews were recorded, transcribed, and submitted for content analysis. All ethical aspects were observed in accordance with Brazilian legislation.

Triangulation involved fusing the data sets for each stage, synthesizing the results in an explanatory structure, and generating a final discussion. During triangulation, inferences and recommendations were extracted from the findings of the combined results for a better understanding of the phenomenon in question [20].

## Results

### Regulation

In Brazil, the Federal Government has exclusive legislative power over professions and has the power and duty to supervise the activities of professions. As state-controlled entities, healthcare professions are regulated by three branches: the legislative branch creates laws for professional practice, the executive branch provides curricula and implements health policies, and the judiciary branch makes decisions in the context of legal disputes. Another important source of regulation is the professional councils, which are federal autarchies, as recognized and authorized by the State, that have the power to supervise and discipline professional practice [21].

Nursing is one of the 24 healthcare professions currently under regulation in Brazil, and the right to exercise this profession is guaranteed by Law No. 7.498/1986 [22], which establishes that nursing can only be practiced by nurses, nursing technicians, nursing assistants, and midwives. It also stipulates that nursing activities can only be performed by persons who are legally qualified and registered with the regional professional council that has jurisdiction in the state where they practice. For nurses, this qualification is granted through a bachelor's degree issued by an institution certified by the Ministry of Education

Nurses’ educational preparation must follow federal guidelines that specify training should prepare students to become professionals with a generalist profile who can cope with the main health problems of the nation [23]. Given the specificities of the SUS, training should include developing skills that apply in different healthcare services, particularly those required by PHC (Table 1).

**Table 1:** Educational guidelines for the Bachelor’s in Nursing degree in Brazil

Graduate profile	Professional with a generalist, humanistic, critical, and reflective training to work at all healthcare levels
Qualifications and emphases	Training must meet the healthcare system’s demands and social health needs and ensure comprehensive, humanized care and quality and emphasize the recognition of the right to health in technical and scientific performance and in health promotion, maintenance, prevention, protection, and recovery
General skills and abilities	Healthcare; decision-making; communication; leadership; administration and management; permanent education; and contextualized technical/scientific, ethical/political, and social/educational skills
Specific skills corresponding to primary care	- Acknowledge health as a right and act in a way to guarantee comprehensive care, understood as the coordinated, continuous set of preventive and curative individual and collective actions and services required for each case at all levels of the system’s complexity
	- Be active in comprehensive healthcare programs for children, adolescents, women, adults, and the elderly
	- Be able to diagnose and resolve health problems, communicate, make decisions, intervene in the work process, work in a team, and face constantly changing situations
	- Respond to regional health specificities through strategically planned interventions in health promotion, prevention, and rehabilitation, paying full attention to the health of individuals, families, and communities
	- Assume an ethical, humanistic, and social commitment to multiprofessional healthcare work
	- Act in different scenarios of professional practice based on the premises of clinical and epidemiological models
	- Identify the population’s individual and collective health needs, their conditions, and determinants
	- Intervene in the health–disease process and take responsibility for the quality of nursing assistance/care in its different healthcare levels with health promotion, prevention, protection, and rehabilitation actions from the perspective of comprehensive care
Curricular contents	Biological and Social Foundation of Nursing (Morphology, Physiology, Pharmacology, Pathology, Cellular and Molecular Biology, Nutrition, Public Health and Environmental Health/Ecology); Human Sciences (Anthropology, Philosophy, Sociology, Psychology, Communication, and Education); Fundamentals of Nursing; Nursing Assistance; Nursing Administration; Nursing Education
Internships	The minimum workload for the supervised curricular internship must total 20% of the total course load. In addition to the theoretical and practical content developed throughout the nurses’ training, nursing programs are required to include in their curriculum a supervised internship in general and specialized hospitals, outpatient clinics, primary care service network, and communities
Duration	Nursing programs must have a minimum course load of 4,000 h and a minimum limit of five years

According to Law 7.498/1986 [22], the nurse is responsible to perform all nursing activities, while managerial practices and activities of greater clinical complexity are exclusive to them. With the exception of these exclusive activities, this legal provision does not detail the actions included within the nurse’s scope of practice. This may be because part of a nurse’s functions has been historically legitimized, as it is one of the first healthcare professions to be regulated in Brazil. Meanwhile, many practices, usually reserved for nurses, have been incorporated by other professions (such as nutrition and psychology), thus resulting in a need to review nurses' attributions.

This dynamic is permanent in the world of healthcare professions, largely in part because professional councils have normative and regulatory competence that allows them to define the activities that can be performed by their professionals [21]. Between 1975 and 2020, COFEN published more than 300 provisions that ensure, authorize, or prohibit practices. These provisions often trigger jurisdictional disputes over particular or exclusive acts, especially in regard to medicine, which has solid ties with parliamentary leaders and constantly attempts to restrict other professions’ scope of practice to preserve its exclusive practice areas.

In the context of PHC, nursing activities must take into account the epidemiological aspects and guidelines for the healthcare practices set forth in Law 7.498/1986 [22] and in the Brazilian primary care policy [24] that describes the general duties of those who comprise the multiprofessional healthcare teams. More detailed functions are contained in guidelines, manuals, and thematic journals that specify care for particular groups (e.g., women's health, children's health, chronic diseases) and are updated periodically based on new evidence. From this perspective, nurses have legal prerogatives for expanded assistance in Brazil: in PHC, they can request complementary tests, prescribe medication, and refer patients to other professionals and services. However, in the local context, municipalities define the scope of this assistance based on technical norms or specific nursing protocols.

Table 2 presents a summary of the set of authorized practices for Brazilian nurses.

**Table 2:** Synthesis of the set of permitted and prohibited practices for Brazilian nurses based on legal provisions

Provision	Practices
	Authorized practices
Law No. 7.498/1986—Professional Practice Law	*Nurse's exclusive practices*
	a) Manage entities and head nursing services and units
	b) Plan, organize, coordinate, execute, and evaluate nursing assistance services
	c) Consult, audit, and issue opinions on nursing matters
	d) Nursing consultations
	e) Prescribe nursing assistance
	f) Direct nursing care for critically ill patients at risk of death
	g) More technically complex nursing care that requires scientific knowledge and the ability to make immediate decisions
	*The nurse as a member of the healthcare team (shared practices)*
	a) Participates in the planning, execution, and evaluation of healthcare programs and plans
	b) Prescribes drugs provided for in public healthcare programs and routinely approved by the healthcare institution
	c) Prevents and systematically controls nosocomial infection, communicable diseases in general, and harm that may be caused to patients
	d) Nursing care for pregnant women, prenatal and postnatal care
	e) Monitor progress and labor and delivery
	f) Delivery without dystocia
	g) Education that aims to improve the population’s health
	*The obstetric nurse as a member of the healthcare team (shared practices)*
	a) Provides assistance in prenatal care and in normal childbirth
	b) Identifies obstetric dystocia and takes measures until the physician’s arrival
	c) Performs episiotomy and episiorrhaphy and applies local anesthesia, when necessary
Resolutions issued by the Federal Nursing Council	*Nurse’s exclusive practices within the nursing team*
	a) The nurse can request routine and complementary exams
	b) Collect material for oncotic colpocytology by Pap smear
	c) Classify risk and prioritize assistance in urgent care services
	d) Insert urinary catheters
Guidelines, manuals, and thematic journals orienting primary care, issued by the Ministry of Health	*Nurse’s specific practices in primary care depending on local clinical protocols*
	a) Provide healthcare to individuals and families linked to teams in the unit, at home, and in other community spaces
	b) Perform nursing consultations and procedures, request complementary exams, prescribe medications according to protocols, clinical and therapeutic guidelines, or other technical norms established by the federal, state, or municipal administrator, observing the legal provisions for the profession
	c) Receive patients with qualified listening and risk classification
	d) Conduct group activities and refer patients to other services, when necessary
	e) Perform prenatal consultations for low-risk pregnant women, alternating with the presence of the physician
	f) Perform rapid tests
	g) Perform clinical breast examinations and collections for cervical cytopathological examinations
	h) Family planning and prescription of contraceptive methods (except definitive ones)
	i) Normal delivery without dystocia
	Prohibited practices
Nursing Code of Ethics and resolutions issued by the Federal Nursing Council	a) Deny nursing care in urgent, emergency, epidemic, disaster, and catastrophic situations
	b) Perform surgical acts, except in emergency situations or in those expressly authorized by law, provided that the nurse has the necessary technical/scientific skills
	c) Prescribe medications that are not established in public healthcare programs and/or in routines approved by a healthcare institution, except in emergency situations
	d) Provide services that are, by their nature, the responsibility of another professional, except in emergencies or those expressly authorized by current legislation
	e) Delegate exclusive activities to other members of the healthcare team
	f) Apply sutures, except in urgent cases where there is an imminent and serious risk of life
	g) Comply with a remote medical prescription or after the expiration date

### Practice

**Fig. 1:**
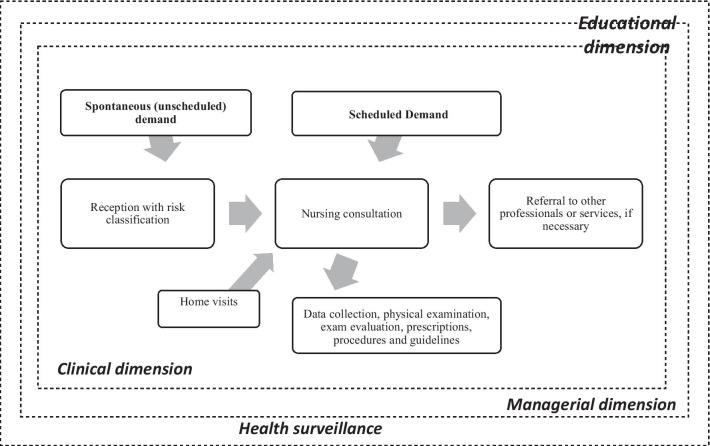
Dimensions of practices performed by Brazilian nurses in primary healthcare

The practices performed by nurses in PHC can be divided into four interdependent dimensions: clinical, managerial, health surveillance, and educational. The investigative dimension was not evidenced in the literature (Fig. 1).

The managerial dimension mainly includes the practices of organizing, planning, and supervising the actions of technicians, assistants, and community agents; holding and participating in meetings; and setting agendas. In general, nurses are also the managers of the health units, and this dimension occupies a large portion of the nurses' working time, reinforcing the idea that much of what nurses do is invisible [25–31].

The purpose of health surveillance is to constantly observe and analyze the population's health status to control health determinants, risks, and harm. This dimension includes actions to promote, prevent, and control illnesses and vulnerabilities over the area of operation, such as active searches, notification of events in public health interest, and vaccination campaigns [26–33]. The educational dimension, which is part of the other dimensions, encompasses health education actions for individuals, families, and groups; permanent education for other team professionals; and student mentorship [25–35].

Clinical practices—care provided directly to the user—are most performed by nurses and occur mainly in the context of spontaneous demand and nursing consultations, either in the unit or during home visits. Nursing consultations constitute a strategy that favors performing the actions prescribed in programs that integrate PHC. Moreover, these actions are the main reasons why nurses provide care, such as management of chronic non-communicable diseases, sexual and reproductive health, women’s health, and prenatal care [25–44]. In this dimension, the most common technical procedures include the administration of vaccines and medications, the collection of oncotic colpocytology (Pap smear) and test materials, and the application of dressings and glucose tests. The most common prescriptions are for dressings, and—according to clinical protocols—for medication, supplements, and laboratory tests, especially within prenatal care and diabetes and hypertension management [27–30, 35, 36, 39–46].

A study [40] conducted on more than 2500 nurses working in PHC found that these nurses knew how to perform more activities in addition to those that they carried out in their workplace. The reasons for their limited work practice were the lack of municipal clinical protocols and/or the existence of restrictive measures by the professional council. The nurses who performed exclusive medical activities—abscess drainage (30%), sutures (5%), and local anesthesia (6%)—did so either because there was a lack of doctors in the units or because the doctors did not have the technical skills to perform them [29, 39, 40].

### Education

In terms of the practices taught in undergraduate nursing programs, we determined that students are prepared to carry out various actions that are legally and socially recognized as nurse’s activities. Meanwhile, the practices that nurses are allowed to perform, but are historically understood to be the physician’s responsibility, were mostly classified as capacities that were not covered or only partially covered by programs (Table 3). In face-to-face interviews, the evaluation of the last set of actions, especially prescribing medication, was preceded by justifications based on the perception that these functions go beyond the nurse’s scope of practice.

“We do not teach how to prescribe any medication, not even those provided for in the protocols. It falls under the physician’s responsibility [...]. The nurse performs diagnostic tests because it is technical, but interpreting and making diagnoses does not fall under the nurse’s responsibility either.” (Southern macroregion)

“There is the issue of primary care protocols that dictate what nurses can do. But, seriously, the nurse has to do what a nurse has to do. We cannot be concerned with what the other person does. There is no point in wanting to prescribe medication if one does not even know how to make a nursing diagnosis.” (Southeastern macroregion)

**Table 3:** Percentage of nursing programs according to the preparation of students for the development of actions (*n* = 110)

Actions and procedures	Prepare	Partially prepare	Do not prepare
*Actions legally assigned and historically recognized as nurses' activities*			
Insert nasogastric, nasoenteric, and urethral intubation	99.1	1.0	0.0
Apply dressings	99.1	1.0	0.0
Administer medications	99.1	0.9	0.0
Conduct health education groups	98.2	0.9	0.9
Execute health promotion actions	97.2	2.8	0.0
Administer vaccines	96.7	2.1	1.1
Plan and execute vaccination campaigns	96.3	2.8	1.0
Perform compulsory notifications	95.7	4.3	0.0
Execute family planning	95.3	3.8	1.0
Perform Pap smear	94.5	3.7	1.9
Perform pediatric consultation	93.6	4.6	1.8
Interpret Pap smear	91.5	3.2	5.3
Perform low-risk prenatal care	90.9	9.1	0.0
Receive patients with risk classification	87.2	11.0	1.8
Prescribe dressings	83.9	10.7	5.4
Perform electrocardiograms	83.2	9.3	7.5
Insert peripheral venous catheters	81.3	10.2	8.4
Perform artery punctures	76.3	10.7	12.9
Average	**92.2**	**5.1**	**2.7**
*Actions legally permitted for nurses but historically recognized as exclusive to the physician*^*a*^			
Interpret laboratory tests	75.2	22.0	2.7
Interpret imaging tests	53.2	25.6	21.1
Request laboratory tests	47.7	29.4	22.9
Prescribe medications provided for in institutional protocols	46.7	46.7	6.7
Perform normal deliveries	42.1	18.7	39.2
Request imaging tests	23.1	24.1	52.7
Prescribe anthelmintics	19.6	15.9	64.5
Prescribe antifungals	13.1	9.8	77.1
Prescribe anti-inflammatory drugs	12.0	6.5	81.5
Prescribe antibiotics	9.3	15.9	74.7
Average	**34.2**	**21.5**	**44.3**
*Actions exclusive to the physician*			
Perform abscess drainage	47.1	14.1	38.7
Communicate nosological diagnoses to patients^b^	26.9	12.9	60.2
Prescribe medications for people with chronic illness diagnosed by the physician	17.6	18.5	63.9
Renew medical prescriptions	12.8	5.3	81.9
Apply sutures^c^	11.1	17.6	71.3
Perform local anesthesia^c^	9.7	7.5	82.8
Confirm death	8.7	7.6	83.7
Prescribe psychoactive drugs	5.4	3.3	91.3
Make nosological diagnoses^b^	4.3	2.1	93.6
Average	**16.0**	**9.9**	**74.2**

^a^Legally permitted by the Federal Nursing Council and the Ministry of Health but subject to the local administrator’s authorization through the creation of nursing protocols

^b^According to the Law on the Professional Practice of Medicine, these practices are not exclusive to the physician, but judicial decisions have ruled in favor of the understanding that the physician is the only professional legally authorized to perform diagnoses. Although it is not exclusive, it can only be shared with other professions by way of federal laws and never by council resolutions or municipal or state laws. Within PHC, these actions can be performed by nurses in services where clinical nursing protocols are in place, but they are limited to common diseases that are not serious (e.g., worm infestations, dermatitis, sexually transmitted infections)

^c^Obstetric nurses can perform these actions within the scope of gynecological assistance (perform episiotomy and episiorrhaphy with the application of local anesthesia)

Moreover, we found that students are not prepared to perform exclusive medical activities. Nevertheless, directors recognized that during educational internships, students accompany nurses who performed such activities, especially in rural areas.

"In the countryside, many nurses perform medical actions in the absence of a physician." (Midwestern macroregion)

“My students accompany nurses who prescribe medication, apply sutures. And they will surely have to perform these procedures out of necessity after they graduate. Will they see a person with tuberculosis or pneumonia and, knowing the appropriate medication, not prescribe it, because it is not within their responsibility? Can I characterize that as what, neglect?” (Northern macroregion)

## Discussion and conclusion

The process of introducing APN in Brazil remains in the initial stages of discussion. In addition, the first studies on the subject remain in the early stages of development, the results of which may define the APN model for implementation. Jhpiego [47] proposes initiating the operationalization of the model by defining its scope of practice, which includes describing the professional’s activities, responsibilities, and level of authority based on a situational analysis of the demands of the healthcare system. Subsequently, the skills required for safe, effective practice and the policies that ensure professional autonomy must be defined.

In Brazilian PHC, common illnesses, which are generally benign, self-limiting, and minor, and social illnesses, occur more frequently in a community. Despite this plurality, demand is concentrated on a few problems or reasons for consultation [48], the most frequent being chronic diseases, especially non-insulin-dependent diabetes and uncomplicated arterial hypertension; family planning; prenatal care; and health maintenance/disease prevention [48–51]. These demands require developing nursing actions that align with the skill set in the curricular guidelines and are anchored in the scope of practice authorized by the legislation and primary care programs guidelines.

This study demonstrates that these demands correspond with the main reasons for the nurse’s care in PHC. They perform a set of clinical, managerial, educational, and health surveillance activities, including advanced practices, according to international models [10–12]. Although to a lesser extent, nurses also perform exclusive medical practices in the absence of a physician in health units. Furthermore, this study indicates that most undergraduate programs do not fully prepare students to adequately execute advanced tasks. These results corroborate with the findings of other studies conducted on the nursing field [40, 52].

This gap in the training process can be attributed to several factors, such as: (a) the lack of articulation between educational institutions and health services, resulting in curricula that do not correspond to the training demands required by the health context [17, 18]; (b) although extensive, the workload of bachelor's degree courses is insufficient to promote the development of all the skills required by the health system [18, 53]; (c) advanced practice nursing is a little explored and controversial subject in Brazil. The implementation of these practices is not unanimous among nurses, and it is poorly understood by other professionals, health managers, nursing professors and SUS users [19, 54]; (d) the training regulations are generic, allowing different interpretations, and are obsolete, as they no longer reflect the social and health needs of Brazil [18, 53]; (e) some nurses and nursing professors believe that the profession has distanced itself from its real attributions in the care process, and it is not up to them to incorporate other activities [19]; (f) the bachelor's degree courses in nursing are mostly private (90%), whose teaching has been characterized by the precariousness of practical activities. In this aspect, there is a differentiated training in public and private schools that neither serve the interests of the profession nor the training of professionals for the SUS [53].

Although it is verified that there has been an increase in nurses' autonomy within the PHC over the last decades, notably due to the expansion of their clinical performance supported by legal documents, their work is still technically subordinate to that of the physician and is thus socially understood [55]. This finding reaffirms that the training model is still centered on clinical specialties and guided by the logic of "professional tribalism”,^2^ as opposed to the logic of training and work in PHC, which is based on health needs population, interdisciplinarity and professional collaboration [56, 57]. All these factors, to a lesser or greater degree, are reflected in the curricula and pedagogical practices of bachelor's degree in nursing.

Despite this, advanced practices seem to already be occurring in Brazil. There are national documents that ensure and authorize nursing graduates to conduct these practices, thus rendering the municipalities to prepare the appropriate clinical guidelines for local needs. The guidelines must comply with the legal and ethical principles of the profession and SUS rules and regulations, consider the best available evidence, and enable professional autonomy. Furthermore, once deficiencies in the training process have been verified, the current education model should undergo reforms to incorporate the skills compatible with the regulated advanced practices. There is already a need to expand the curricular contents of pharmacology, pathophysiology, and evidence-based practice [52, 58].

Immediately, additional training through interprofessional continuing education processes is recommended for professionals who already work in PHC, to ensure the quality of care and the professional's safety in their performance of advanced practices. It is also imperative to update the normative acts that regulate education and the scope of practice, as they are outdated and too general in terms of describing the skills and the scope of advanced practices (Table 4).

**Table 4:** Convergent matrix of the research results

Components	Results	Inferences	Recommendations
Regulation *Objective*: to identify legally authorized practices *Method:* document analysis	a) Curricular guidelines: comprehensive, indicate skills and abilities for primary care practices b) Normative documents of the Legislative and Executive branches and the professional council: regulate advanced practices in the context of primary healthcare (PHC) (nosological diagnoses of common and non-serious diseases, drug prescriptions, requests and interpretations for diagnostic tests, referrals) but are dependent on clinical protocols established by local administrators	a) The Professional Practice Law is outdated (it was created before the Brazilian Unified Health System (SUS)) and does not fully detail the activities that nurses can perform. The gaps in the legislation focus on the appropriation of tasks by other professions; excessive publications of regulations by professional councils that revoke, include, or ratify tasks; legal disputes with other categories, especially with physicians; insecurity in the professionals who provide care; limited social perception of the nurse’s scope of practice b) The practices performed by the nurse respond to the main healthcare demands of PHC c) The advanced practices prescribed in the legislation and performed by PHC nurses correspond to those defined by other countries d) The nurse’s autonomy is limited in places where there are no established clinical protocols, which points to nurses’ underutilization e) Exclusive medical acts are performed by nurses in places where there are no doctors f) Advanced practices have already been implemented in the context of Brazilian PHC, so it is not necessary to introduce the APN as a new professional category	a) Implement clinical protocols in all municipalities based on local needs b) In-service training for advanced practices c) Reforms in the training/education model that ensure the acquisition of skills for advanced practices d) Update the Professional Practice Law so that it considers incorporating the premises of the SUS, the content of the different normative acts issued by the professional council, and the details of the set of authorized activities for nurses
Practices *Objective:* to identify the practices performed in the context of PHC *Method:* systematic review	a) Managerial, educational, clinical, and health surveillance practices b) Advanced practices based on clinical protocols: nosological diagnoses of common and non-serious diseases, drug prescriptions, requests and interpretations for diagnostic tests, referrals c) Exclusive medical practices: sutures, abscess drainage, application of local anesthesia		
Education *Objective:* to identify the practices taught in undergraduate nursing programs *Method:* exploratory research	a) The program prepares students for practices legally and historically recognized as nurses’ activities b) Partly prepares for advanced practices c) Does not prepare for exclusive medical practices d) Education administrators do not recognize advanced practices as part of the nurse's scope of practice		

**Fig. 2:**
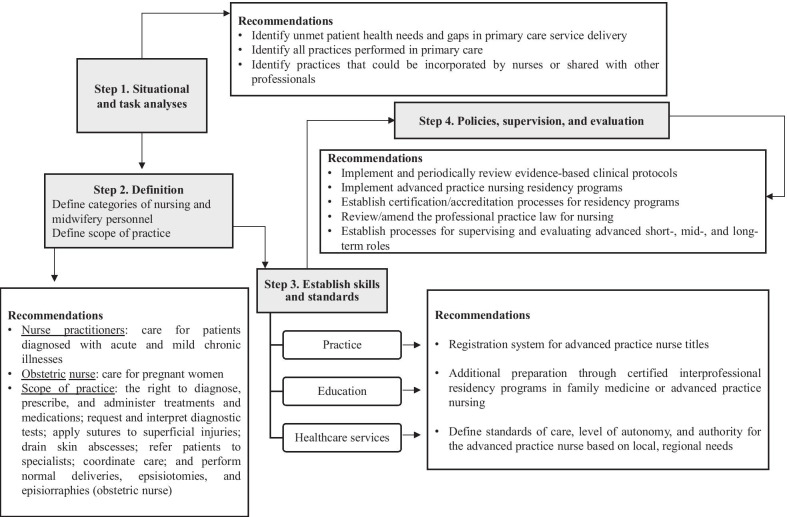
Steps and recommendations for operationalizing the Brazilian advanced practice nurse model

If Brazil decides to introduce the international APN model, either by safeguarding current advanced practices or by expanding them, the process would be more arduous and time-consuming. It would require reforming the current model of professional regulation in order to comply with the countless recommendations that sustain APN characteristics, such as additional educational preparation in certified programs at the professional master's degree level, protected titles, and specific APN regulations [8, 12]. Figure 2 proposes the steps to operationalize this APN model.

Unlike international recommendations, we do not believe that the professional master's degree is the most appropriate level of training in the Brazilian context. These programs are found mostly in large urban centers and are strongly influenced by academic models that contrast with the proposal to train nurses in the exercise of professional, transformative practice [59]. We suggest seeking additional training in lato sensu graduate programs,^3^ especially in interprofessional family medicine residency programs.

Regardless of the adoption of the APN model, other future objectives in Brazil include fortifying education and interprofessional work and permanent education actions, wage reform, investment in infrastructure and working conditions, and greater control over the formation and quality of undergraduate programs. Moreover, a greater governmental role is necessary in the regulation of healthcare professions, specifically to mitigate weaknesses of laws on the exercise of professions and to resolve corporate clashes over disputes regarding scope of practice [60].

Expanded, fluid, and flexible regulatory legislation could be defined based on inter-professionalism and task sharing, thus establishing common goals for all healthcare professions and specific regulations for a given profession. This model could provide flexibility in the division of healthcare work by adopting public interest as its guiding principle, rather than the profession’s organizations, its self-regulation, or the monopoly imposed by professional categories.

## Supplementary Information

**Additional file 1:** Scoping review. Detailed information about the scoping review. **Table S1.** Terms selected for research. **Table S2.** Search strategies. **Table S3.** Characterization variables in the set of publications. **Table S4.** Indexing data, aims, and methods of the set of publications. **Table S5.** Set of practices carried out by Primary Health Care nurses. **Table S6.** Technical procedures and prescriptions carried out by Primary Health Care nurses. **Figure S1.** Flowchart of the refinement process.

## Data Availability

All data analyzed during this study are included in this published article and its supplementary information files.

## Abbreviations

APN: Advanced practice nurse
COFEN: Federal Nursing Council (Conselho Federal de Enfermagem, in Portuguese)
PHC: Primary healthcare
SUS: Unified Health System (Sistema Único de Saúde, in Portuguese

## Footnotes

1 This is the professional nursing council in Brazil—an organization created by federal law for the purpose of regulating, standardizing, and supervising the profession it represents.

2 Tendency of each profession to act in isolation. It is the result of the culture of separate training, with an emphasis on the development of specific skills, which ends up building very rigid professional identities and which, historically, has been configured as a barrier to communication between professionals of different categories.

3 In Brazil, there are two types of postgraduate; lato sensu, which encompasses specialization and residency programs in one area of study, mostly addressed to professional practice, and stricto sensu, which means a Master or Doctorate, encompassing broader and profound activities of scientific research.
