# Supplementary material for: Situational analysis and reflections on the introduction of advanced practice nurses in Brazilian primary healthcare
Source: Hum Resour Health. 2021 Jul 22;19:90. doi: 10.1186/s12960-021-00632-w (PMC8296671; doi:10.1186/s12960-021-00632-w)
Supplement: Supplementary file 1 — Additional file 1: Scoping review. Detailed information about the scoping review. Table S1. Terms selected for research. Table S2. Search strategies. Table S3. Characterization variables in the set of publications. Table S4. Indexing data, aims, and methods of the set of publications. Table S5. Set of practices carried out by Primary Health Care nurses. Table S6. Technical procedures and prescriptions carried out by Primary Health Care nurses. Figure S1. Flowchart of the refinement process. [file 12960_2021_632_MOESM1_ESM.pdf]

ADDITIONAL FILE I – SCOPING REVIEW

Aim
To identify and map the literature on the practices developed by nurses in the context of Primary Health Care (PHC) in Brazil.

Study design
A scoping review of the national literature on the topic in perspective was carried out, namely: the practices developed by nurses in the context of PHC in Brazil. Scoping review was chosen, instead of other types of knowledge synthesis, because it follows a systematic approach to map the evidence and be considered exploratory and appropriate when the purpose is to examine the extent and nature of the productions, clarify concepts, identify potentialities, and systematize findings on a given topic that is still little explored[1].

Research protocol
The research was guided by *Preferred Reporting Items for Systematic reviews and Meta-Analyses extension for Scoping Reviews* (PRISMA-ScR)[1], a guideline that describes a minimum set of items to be considered in the development of scope review reports, ensuring its methodological transparency. Regarding the method, the PRISMA-ScR guides the achievement of the stages of the review, which includes: the outline of the guiding question; definition of the eligibility criteria; selection of information sources; definition of search strategies and selection process; analysis; and synthesis of the results.

Guiding question
The PCC approach[2] (P = population; C = concept; C = context) was followed to formulate the research question: What are the practices performed by nurses in the context of PHC in Brazil? In this case, nurses, practices and PHC in Brazil were considered population, concept, and context, respectively.

Eligibility criteria
Scope and types of evidence sources
All primary studies focused on the topic, published in Portuguese, English or Spanish, were included in the sample. No time limits and methodological approaches were adopted. Abstracts, editorials, reviews, letters, interviews, videos, and redundant publications were excluded.

Population, concept, and context
Considering the PCC mnemonic, publications that included nurses working in Brazilian PHC, of any gender, age, or working time were included. Additionally, for inclusion, studies needed to report at least one practice (action, activity, intervention) performed by nurses in their daily work.

Information sources and search strategies
To define search strategies, a limited mapping was initially carried out in the Virtual Health Library (VHL), based on the strategy “nursing AND primary care AND Brazil”. This initial search was followed by an analysis of the words of the text contained in the title and keywords of the articles retrieved and published in the last two years. From this step, it was possible to select a set of descriptors corresponding to the thesauri adopted by the VHL and Medical Literature Analysis and Retrieval System Online (MEDLINE), and non-indexed terms (Table 1). These were combined by the Boolean operators AND, OR and NOT, constituting different search strategies according to the researched database (Table 2).
Subsequently, the systematized searches were carried out in March 2021, in the databases of Latin American and Caribbean Literature in Health Sciences (LILACS); Scientific Electronic Library Online (SciELO); MEDLINE via PUBMED; and Cummulative Index to Nursing and Allied Health Literature (CINAHL).

[1] Tricco AC, Lillie E, Zarin W, O’Brien KK, Colquhoun H, Levac D. et al. PRISMA Extension for Scoping Reviews (PRISMA-ScR): Checklist and Explanation. Ann Intern Med. 2018;169(7):467-73. https://doi.org/10.7326/M18-0850
[2] Joanna Briggs Institute. The Joanna Briggs Institute reviewers' manual 2015: methodology for JBI scoping reviews. Adelaide: Joanna Briggs Institute; 2015

Table S1. Terms selected for research

Language	Search terms		
	**Population**	**Concept**	**Context**
English	nurses OR nursing OR nurse midwives OR primary care nursing OR primary nursing OR family nurse practitioners OR community health nursing OR public health nursing OR family nursing	advanced practice nursing OR nurse's role OR professional practice OR professional role OR nursing care OR scope of practice	primary health care OR family health strategy OR family health<br><br>AND brazil
Portuguese	enfermeiras e enfermeiros OR enfermagem OR enfermeiras obstétricas OR enfermagem de atenção primária OR enfermagem primária OR enfermeiras de saúde da família OR enfermagem em saúde comunitária OR enfermagem em saúde pública OR enfermagem familiar	prática avançada de enfermagem OR papel do profissional de enfermagem OR prática profissional OR cuidados de enfermagem OR âmbito da prática	atenção primária à saúde OR estratégia saúde da família OR saúde da família<br><br>AND brasil

Table S2. Search strategies

Source	Search strategies
**Lilacs**	enfermeiras e enfermeiros OR enfermagem OR enfermeiras obstétricas OR enfermagem de atenção primária OR enfermagem primária OR enfermeiras de saúde da família OR enfermagem em saúde comunitária OR enfermagem em saúde pública OR enfermagem familiar [Palavras] AND prática avançada de enfermagem OR papel do profissional de enfermagem OR prática profissional OR cuidados de enfermagem OR âmbito da prática [Palavras] AND (atenção primária à saúde OR estratégia saúde da família OR saúde da família) AND brasil [Palavras]
**SciELO**	((enfermeiras e enfermeiros) OR (enfermagem) OR (enfermeiras obstétricas) OR (enfermagem de atenção primária) OR (enfermagem primária) OR (enfermeiras de saúde da família) OR (enfermagem em saúde comunitária) OR (enfermagem em saúde pública) OR (enfermagem familiar)) AND ((prática avançada de enfermagem) OR (papel do profissional de enfermagem) OR (prática profissional) OR (cuidados de enfermagem) OR (âmbito da prática)) AND ((atenção primária à saúde) OR (estratégia saúde da família) OR (saúde da família)) AND (brasil)
**Medline**	(((nurses OR nursing OR nurse midwives OR primary care nursing OR primary nursing OR family nurse practitioners OR community health nursing OR public health nursing OR family nursing[MeSH Terms]) AND (advanced practice nursing OR nurse's role OR professional practice OR professional role OR nursing care OR scope of practice[MeSH Terms])) AND (primary health care OR family health strategy OR family health[MeSH Terms])) AND (brazil[Title/Abstract]) Filters: Journal Article, Humans, English, Portuguese, Spanish
**Cinahl**	TI (nurses OR nursing OR nurse midwives OR primary care nursing OR primary nursing OR family nurse practitioners OR community health nursing OR public health nursing OR family nursing) AND (advanced practice nursing OR nurse's role OR professional practice OR professional role OR nursing care OR scope of practice) AND (primary health care OR family health strategy OR family health) AND brazil

A third step consisted of verifying the reference list of the selected studies (cross-reference), in order to identify material of interest for this study - not recovered in the previous phase - which were accessed by the electronic databases.

Selection, analysis, and results synthesis process

The selection of publications was carried out in three stages: screening, in which duplicate studies were excluded; eligibility, stage in which the titles, abstracts and keywords of the records were read by two independent reviewers, applying previously defined criteria; and consensus, when the remaining publications were recovered and submitted to full reading guided by a script, by the two reviewers who, together, determined the inclusion or exclusion of the selected material (Figure 1).

The analytical reading of the studies sought to identify the set of practices performed by nurses in the context of PHC. The data were compiled in Excel spreadsheets and text files and subjected to the usual techniques of descriptive analysis and content analysis. In the end, a synthesis of knowledge was produced from thematic categories.

Figure S1. Flowchart of the refinement process

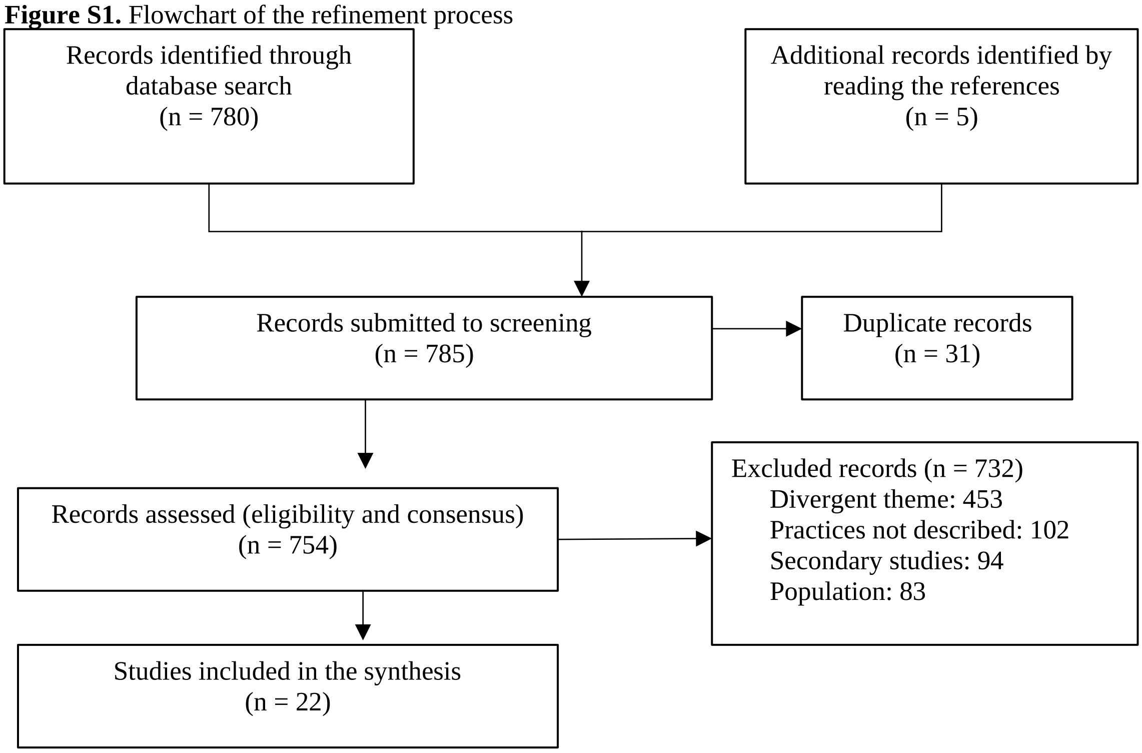

Characteristics of publications

Table S3. Characterization variables the set of publications

Characterization variables	N	%
**Year**		
2009	1	4.5
2011	1	4.5
2013	2	9.1
2014	1	4.5
2015	3	13.6
2016	1	4.5
2017	5	22.7
2018	4	18.2
2019	3	13.6
2021	1	4.5
**Language**		
More than one language	13	59.1
Portuguese	8	36.4
English	1	4.5
**Type of publication**		
Journal article	18	81.8
Thesis	2	9.1
Dissertation	1	4.5
Research Report	1	4.5
**Methodological Approach**		
Qualitative	14	63.6
Quantitative	8	36.4
**Data collection method**		
Interviews	8	36.4
Interviews and observation	4	18.2
Observation	4	18.2
Self-administered questionnaire	3	13.6
Online survey	2	9.1
Focus group	1	4.5

Table S4. Indexing data, aims, and methods of the set of publications.

Year	Type	Language	Authors	Title	Aim	Sample of nurses	Methods	Data collect
2021	Article	Portuguese	Mendes M et al.	Práticas da enfermagem na estratégia saúde da família no Brasil: interfaces no adoecimento	To identify Nursing practices in the Family Health Strategy in Brazil and interfaces in the illness of these professionals.	45	qualitative and exploratory	interviews and observation
2016	Article	Portuguese and English	Bonfim D et al.	Time standards of nursing in Primary Health Care: an observational study	To determine time standards for interventions and activities conducted by nursing professionals in Family Health Units in Brazil to substantiate the calculation of work force	47	observational and quantitative	observation
2017	Thesis	Portuguese	Lemos PFS	As ações de saúde realizadas por enfermeiros e o uso de protocolos clínicos e diretrizes terapêuticas no contexto atual da Estratégia Saúde da Família no município do Rio de Janeiro	To analyze the health actions performed by nurses and the use of clinical protocols and therapeutic guidelines in the current context of the Family Health Strategy in the city of Rio de Janeiro	219	cross-sectional, quantitative, and descriptive	online survey
2018	Article	Portuguese and English	Corrêa VAF et al.	The care of nurses in the Family Health Strategy: practices and theoretical foundation	To analyze the practices of nurses working in the Family Health Strategy in a city of Rio de Janeiro (RJ) and the theoretical foundations that guide them.	12	qualitative and descriptive	interviews
2019	Thesis	Portuguese	Koster I	O exercício profissional da enfermagem no âmbito da Atenção Primária à Saúde no Brasil	To analyze the professional practice of nurses in the context of Primary Heath Care in Brazil, considering the context of changes and extensions of their care practices, in the light of the Sociology of Professions theory, with a view to understanding what elements may provoke changes in the archetype of the profession	10	qualitative and descriptive	focus group
2018	Article	Portuguese and English	Forte ECN et al.	Does the nurses' work change when the Primary Health Care change?	To identify the activities developed by nurses in Basic Health Units, including units that are still under the care model guided by biomedicine as well as units that use the Family Health Strategy model	20	qualitative and exploratory	interviews and observation
2014	Article	Portuguese and English	Paula M et al.	Characteristics of the nurses' work process in the family health strategy	To characterize the work activities undertaken by nurses who work in the Family Health Strategy in a health unit in a city in southern Brazil	4	qualitative and descriptive	observation
2017	Dissertat.	Portuguese	Amoras JAB	Ações de saúde da mulher desenvolvidas pela equipe de enfermagem da Estratégia Saúde da Família no Mato Grosso do Sul	To analyze the actions of women's health developed by the nursing team of the Family Health Strategy in Campo Grande, Mato Grosso do Sul, according to National Primary Health Care Policy	62	cross-sectional, quantitative, and descriptive	self-administered questionnaire
2015	Article	Portuguese and English	Tavares RE and Tocantins FR	Nursing actions in primary care and the control of diseases preventable through vaccines	To discuss the actions performed by nurses in the control and eradication of diseases preventable with vaccines in healthcare setting, according to the National Primary Health Care Policy	10	phenomenological and qualitative	interviews
2017	Article	Portuguese and English	Ferraccioli P and Acioli S	The different dimensions of care in practice held by nurses in primary care	To understand the practices developed in the care provided by nurses in the National Health Programs in a unit of the basic health network in the city of Rio de Janeiro.	8	exploratory and qualitative case study	interviews and observation
2015	Article	Portuguese and English	Acioli S et al.	Care practices: the role of nurses in primary health care	To know the care practices developed by nurses in primary health care in the city of Rio de Janeiro	30	descriptive and qualitative	interviews
2013	Article	Portuguese	Duarte HSJ and Mamede MV	Ações do pré-natal realizadas pela equipe de enfermagem na Atenção Primária à Saúde, Cuiabá	To describe the actions taken by the nursing staff in prenatal city of Cuiabá, MT	74	descriptive and quantitative	observation

2018	Article	Portuguese and English	Becker RM et al.	Nursing care practices for people with chronic noncommunicable diseases	To understand care practices for people with Chronic Noncommunicable Disease, developed by Primary Health Care nurses in a municipality in southern Brazil	23	qualitative; Discourse of the Collective Subject	interviews
2018	Article	Portuguese and English	Vieira DS et al.	Nursing practices in childcare consultation in the Estratégia Saúde da Família	To analyze the care actions performed by nurses during childcare consultations	31	observational and quantitative	observation
2017	Article	Portuguese and English	Girardi SN et al.	Scope of practice in Primary Care: physicians and nurses in five health regions in Brazil	To investigate the scope of practice of physicians and nurses who work in the Primary Healthcare and their main barriers	26	exploratory and qualitative	interviews
2017	Research Report	Portuguese	Pierantoni CR and Girardi SN (Coord.)	Regulação do Trabalho e das Profissões em Saúde	Identify the attributions, competencies and scope of practices different health professions and occupations within the scope of Primary Health Care	2.707	cross-sectional, quantitative, and descriptive	online survey
2011	Article	English	Narchi NZ	Exercise of essential competencies for midwifery care by nurses in São Paulo, Brazil	To analyze the exercise of essential competencies for midwifery care by nurses and/or midwives in the public health system of São Paulo (eastern zone), Brazil	204	descriptive, exploratory, and qualitative	self-administered questionnaire
2018	Article	Portuguese and English	Peduzzi M et al.	Expansion of the interprofessional clinical practice of Primary Care nurses	To analyze the clinical practice of nurses in the interprofessional context of the Family Health Strategy	1	exploratory and qualitative case study	interviews and observation
2015	Article	Portuguese, English, and Spanish	Baptista RS et al.	Prenatal care: essential actions developed by nurses	To investigate the profile, knowledge and practice of nurses who work in low-risk prenatal	15	cross-sectional, quantitative, and descriptive	interviews
2009	Article	Portuguese	Nunes PS et al.	Descrição das práticas dos Enfermeiros da atenção básica direcionadas para idosos diabéticos	To describe the actions that nurses perform with diabetic elderly people and compare them based on the standards issued by the Ministry of Health	42	cross-sectional, quantitative, and descriptive	self-administered questionnaire and observation
2013	Article	Portuguese	Pilger C et al.	Compreensão sobre o envelhecimento e ações desenvolvidas pelo enfermeiro na atenção primária à saúde	To understand the perceptions of nurses about the aging process as well as identify the actions performed by the nurses	10	exploratory and qualitative	interviews
2019	Article	Portuguese and English	Kahl C et al.	Contributions of the nurse’s clinical practice to Primary Care	To understand the repercussions of the nurse’s clinical practice on Primary Health Care	18	qualitative research; Grounded Theory	interviews

Nurses' practices in Primary Care

Table S5. Set of practices carried out by Primary Health Care nurses.

**CLINICS**	**MANAGEMENT**
Humanized reception	Service to users with complaints
Support for exams / procedures	Contact user by phone
Care for patients of spontaneous demand	Supplies control
Risk classification	Program coordination
Communicates nosological diagnosis	Sets consultation schedules
Shared consultation	Dispensing of medicines
Nursing consultation	Documentation
Coordinate care	Issuance of health attestation
Referring users	Professional scale planning
Diagnostic tests interpretation	Unit management
Handles urgencies and emergencies	Leadership
Burns management	Organization of the work process of technicians
Prescriptions	Actions planning
Technical procedures	Data recording in information systems
Home visit	Management of medical agenda
**HEALTH SURVEILLANCE**	Team meetings
Social support for users / family	Administrative routines
Active search	Request ambulances
Registration of families	Request for beds hospital
Control of communicable diseases	Supervision of other professionals
Immunization / vaccination control	
Infection control	
Mapping and territorialization	
Community mobilization	**EDUCATIONAL**
Compulsory notification	Health education (users)
Prevention of diseases	Permanent education (professionals)
Health promotion	Preceptorship (students)

Table S6. Technical procedures, prescriptions and interpretations of diagnostic tests carried out by Primary Health Care nurses within the scope of the clinical dimension.

**Prescriptions**	**Exam interpretation**
Dressings	Electrocardiogram
Imaging exams	Imaging exams
Laboratory tests	Laboratory tests
Drugs	Pap smear
Renew medical prescription	Tuberculin test
Supplements	
Oral rehydration therapy	
Transcribes medical prescription	
Vitamins	
**Technical procedures**	
Apply of medication	Nebulization
Apply of vaccines	Normal childbirth
Apply of local anesthesia	Podiatry
Anthropometry	Tuberculin test
Collection of materials for examination	Pap smear
Dressings	Performs electrocardiogram
Debridement	Suture points withdrawal
Abscess drainage	Apply sutures
Physical exam	Blood glucose test
Orthopedic immobilizations	Neonatal heel prick
Nasogastric, nasoenteric and urethral intubation	Check vital signs

Studies included in the review

Mendes M, Trindade LL, Pires DEP, Martins MMFPS, Ribeiro OMPOL, Forte ECN et al. Práticas da enfermagem na estratégia saúde da família no Brasil: interfaces no adoecimento. Rev Gaúcha Enferm. 2021;42(esp):e20200117. https://doi.org/10.1590/1983-1447.2021.20200117

Bonfim D, Fugulin FMT, Laus AM, Peduzzi M, Gaidzinski RR. Time standards of nursing in Primary Health Care: an observational study. Rev Esc Enferm USP. 2016;50(1):118-26. https://doi.org/10.1590/S0080-623420160000100016

Lemos PFS. Ações de saúde realizadas por enfermeiros e o uso de protocolos clínicos e diretrizes terapêuticas no contexto atual da Estratégia Saúde da Família no município do Rio de Janeiro [thesis]. Rio de Janeiro: Universidade do Estado do Rio de Janeiro; 2017.

Corrêa VAF, Acioli S, Tinoco TF. The care of nurses in the Family Health Strategy: practices and theoretical foundation. Rev Bras Enferm. 2018;71(suppl 6):2767-74. https://doi.org/10.1590/0034-7167-2018-0383

Koster I. O exercício profissional da enfermagem no âmbito da Atenção Primária à Saúde no Brasil [thesis]. Rio de Janeiro: Fundação Oswaldo Cruz; 2019.

Forte ECN, Pires DEP, Scherer MDA, Soratto J. Does the nurses' work change when the Primary Health Care change? Tempus, Actas de Saúde Colet. 2018;11(2):53-68.

Paula M, Peres AM, Bernadino E, Eduardo EA, Sade PMC, Larocca LM. Characteristics of the nurses' work process in the family health strategy. Rev Min Enferm. 2014;18(2):454-70. http://www.dx.doi.org/10.5935/1415-2762.20140034

Amoras JAB. Ações de saúde da mulher desenvolvidas pela equipe de enfermagem da Estratégia Saúde da Família no Mato Grosso do Sul [dissertation]. Campo Grande: Universidade Federal de Mato Grosso do Sul; 2017.

Tavares RE, Tocantins FR. Nursing actions in primary care and the control of diseases preventable through vaccines. Rev Bras Enferm. 2015;68(5):803-9. https://doi.org/10.1590/0034-7167.2015680506i

Ferraccioli P; Acioli S. The different dimensions of care in practice held by nurses in primary care. Rev Fund Care Online. 2017;9(1):28-36. http://dx.doi.org/10.9789/2175-5361.2017.v9i1.28-36

Acioli S, Kebian LVA, Faria MGA, Ferraccioli P, Correa VAF. Care practices: the role of nurses in primary health care. UERJ Nursing Journal. 2015;22(5):637-42. https://doi.org/10.12957/reuerj.2014.12338

Duarte HSJ, Mamede MV. Actions performed by the nursing team in prenatal care at primary health care setting, Cuiabá, Brazil. Cienc Enferm. 2013;19(1):117-29. http://dx.doi.org/10.4067/S0717-95532013000100011

Becker RM, Heidemann ITSB, Meirelles BHS, Costa MFBNA, Antonini FO, Durand MK. Nursing care practices for people with chronic noncommunicable diseases. Rev Bras Enferm. 2018;71(suppl 6):2643-9. http://dx.doi.org/10.1590/0034-7167-2017-0799

Vieira DS, Santos NCCB, Nascimento JA, Collet N, Toso BRGO, Reichert APS. Nursing practices in child care consultation in the Estratégia Saúde da Família. Texto Contexto - Enferm. 2018;27(4):e4890017. https://doi.org/10.1590/0104-07072018004890017

Girardi SN, Stralen ACSv, Lauar TV, Cella JN, Araújo JF, Pierantoni CR et al. Scope of practice in Primary Care: physicians and nurses in five health regions in Brazil. Rev Bras Saude Mater Infant. 2017;17(suppl 1):S171-84. http://dx.doi.org/10.1590/1806-9304201700s100008

Pierantoni CR, Girardi SN (Coord.). Regulação do Trabalho e das Profissões em Saúde [Research Report]. Rio de Janeiro: ObservaRH/IMS/UERJ, EPSM/NESCON/UFMG; 2017.

Narchi NZ. Exercise of essential competencies for midwifery care by nurses in São Paulo, Brazil. Midwifery. 2011;27(1):23-9. http://dx.doi.org/10.1016/j.midw.2009.04.007

Peduzzi M, Aguiar C, Lima AMV, Montanari PM, Leonello VM, Oliveira MR. Expansion of the interprofessional clinical practice of Primary Care nurses. Rev Bras Enferm. 2019;72(Suppl 1):114-21. http://dx.doi.org/10.1590/0034-7167-2017-0759

Baptista RS, Dutra MOM, Coura AS, Stélio SF. Prenatal care: essential actions developed by nurses. Enferm Glob. 2015;14(40):96-111.

Nunes PS, Marques MB, Machado ALG, Silva MJ. Descrição das práticas dos Enfermeiros da atenção básica direcionadas para idosos diabéticos. Cogitare Enferm. 2009;14(4):682-8.

Pilger C, Dias JF, Kanawava C, Baratieri T, Carreira L. Compreensão sobre o envelhecimento e ações desenvolvidas pelo enfermeiro na atenção primária à saúde. Cienc Enferm. 2013;19(1):61-73. http://dx.doi.org/10.4067/S0717-95532013000100006

Kahl C, Meirelles BHS, Cunha KS, Bernardo MS, Erdmann AL. Contributions of the nurse’s clinical practice to Primary Care. Rev Bras Enferm. 2019;72(2):354-9. http://dx.doi.org/10.1590/0034-7167-2018-0348
